# Global characterization of extrachromosomal circular DNAs in advanced high grade serous ovarian cancer

**DOI:** 10.1038/s41419-022-04807-8

**Published:** 2022-04-13

**Authors:** Yixuan Cen, Yifeng Fang, Yan Ren, Shiyuan Hong, Weiguo Lu, Junfen Xu

**Affiliations:** 1 Women’s Reproductive Health Laboratory of Zhejiang Province; Women’s Hospital; School of Medicine, Zhejiang University, Hangzhou, 310006 China; 2 Department of General Surgery, Sir Run Run Shaw Hospital, School of Medicine, Zhejiang University, Hangzhou, 310016 China; 3 Institute of Life Sciences, Chongqing Medical University, Chongqing, 400016 China; 4 Department of Gynecologic Oncology, Women’s Hospital, Zhejiang University School of Medicine, Hangzhou, 310006 China; 5 Cancer Center, Zhejiang University, Hangzhou, 310058 China

**Keywords:** Cancer genetics, Prognostic markers

## Abstract

High grade serous ovarian cancer (HGSOC) is the most aggressive subtype of ovarian cancer and HGSOC patients often appear with metastasis, leading to the poor prognosis. Up to date, the extrachromosomal circular DNAs (eccDNAs) have been shown to be involved in cancer genome remodeling but the roles of eccDNAs in metastatic HGSOC are still not clear. Here we explored eccDNA profiles in HGSOC by Circle-Sequencing analysis using four pairs of primary and metastatic tissues of HGSOC patients. Within the differentially expressed eccDNAs screened out by our analysis, eight candidates were validated by outward PCR and qRT-PCR analysis. Among them, DNMT1^circle10302690-10302961^ was further confirmed by FISH assay and BaseScope assay, as the most significantly down-regulated eccDNA in metastatic tumors of HGSOC. Lower expression of DNMT1^circle10302690-10302961^ in both primary and metastatic tumors was associated with worse prognosis of HGSOC. Taken together, our finding firstly demonstrated the eccDNAs landscape of primary and metastatic tissues of HGSOC. The eccDNA DNMT1^circle10302690-10302961^ can be considered as a potential biomarker or a therapeutically clinical target of HGSOC metastasis and prognosis.

## Introduction

High grade serous ovarian cancer (HGSOC) is the most common and lethal subtype of epithelial ovarian cancer, accounting for nearly 70% death of ovarian cancer [1, 2]. The 5-year survival rate of HGSOC patients at early stages can reach 90%, but only 30% for the patients at advanced stages with widespread metastasis [3]. To elucidate the mechanisms underlying HGSOC metastasis and improve the clinical outcome, efforts have been made to investigate genetic features of HGSOC. HGSOC has been genetically characterized by prevalent TP53 mutation and frequent gene loss (PTEN, RB1, and NF1) or gain (CCNE1, MYC, and MECOM) [4–7]. Other commonly observed genetic alterations in HGSOC include BARD1, BRIP1, CHEK2, MRE11A, MSH6, PALB2, and RAD51C [4, 8]. Al-Kuraya et al. also identified mutations of ARNT, NTRK1, MYH9, PPARG uniquely in the metastatic tissues of HGSOC [9]. However, these gene signatures have not been well implemented clinically [10]. It is still necessary to characterize specific gene expression patterns of HGSOC metastasis for better understanding its machinery, which helps to establish novel clinical diagnostic strategies and to provide potential therapeutic targets for the metastasis treatment.

Located outside chromosomes, a group of novel circular DNAs referred as extrachromosomal circular DNAs (eccDNAs) are generated during DNA damage repair, chromothripsis, and other DNA metabolisms [11–13]. This new circular DNA family has been recently identified in various tissues or cancers by virtue of new technologies such as whole-genome sequencing, ATAC-Sequencing and Circle-Sequencing [14–16]. The previous studies showed that eccDNAs encoding MYC and EGFR oncogenes via self-replication in glioma can be amplified more efficiently than the chromosomal genes [17, 18]. In neuroblastoma, eccDNAs were able to chimerically circularize and reintegrate into linear genomes, resulting in cancer genome remodeling [19]. To date, the roles and functions of eccDNAs in cancers still need more characterization. Moreover, the expression and function of eccDNAs are not yet known in HGSOC primary and metastatic tissues.

Here, we characterize the eccDNA expression profiles of primary and metastatic tissues of HGSOC, and identify a novel eccDNA named DNMT1^circle10302690-10302961^, which has been validated as the most significantly down-regulated eccDNA in metastatic tumors compared to primary tumors of HGSOC. Additionally, we determine the clinical value of DNMT1^circle10302690-10302961^ in a cohort of HGSOC tissues. The decrease of DNMT1^circle10302690-10302961^ is associated with a poor prognosis for HGSOC patients. Taken together, our work demonstrate an eccDNA signature of HGSOC, which can be considered as a potential diagnostic strategy for metastasis prevention, and a specific eccDNA DNMT1^circle10302690-10302961^, which can be a credible clinical therapeutic target for HGSOC metastasis treatments.

## Results

### Detection of eccDNAs in HGSOC samples by Circle-Sequencing analysis

**Fig. 1:**
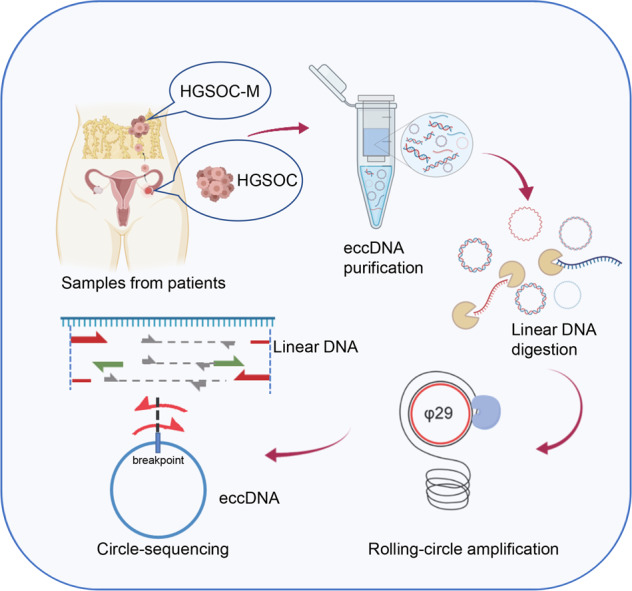
A schematic to illustrate Circ-Seq. Paired primary tissues (HGSOC 1-4) and metastatic tissues (HGSOC-M 1-4) of HGSOC were obtained from four HGSOC patients. EccDNAs were separated, purified and rolling-circle amplified for Circ-Seq. Detection of eccDNA was based on soft clipped reads (Red arrows, soft-clipped reads; Grey arrows, concordant reads; Green arrows, discordant reads).

To detect eccDNAs in the paired primary and metastatic tissues (HGSOC-M) of HGSOC, we conducted Circle-Sequencing analysis with four pairs of specimens (Supplementary Table 1). The main steps of this novel approach were listed in Fig. 1. Briefly, the total circular DNAs were separated using column chromatography. The separated samples were treated with endonucleases and restriction exonucleases to remove mitochondrial circular DNA and residual linear chromosomal DNA. The purified eccDNAs were then replicated by the rolling-circle amplification using φ29 DNA polymerase. The amplified products were finally analyzed using high-throughput sequencing mapped to the reference genome (UCSC hg19). In the present study, 194955 eccDNAs were identified by Circle-Map software in all samples [20]. Our results showed an abundant eccDNA expression in HGSOC tissues.

### Features of eccDNAs detected in HGSOC samples

**Fig. 2:**
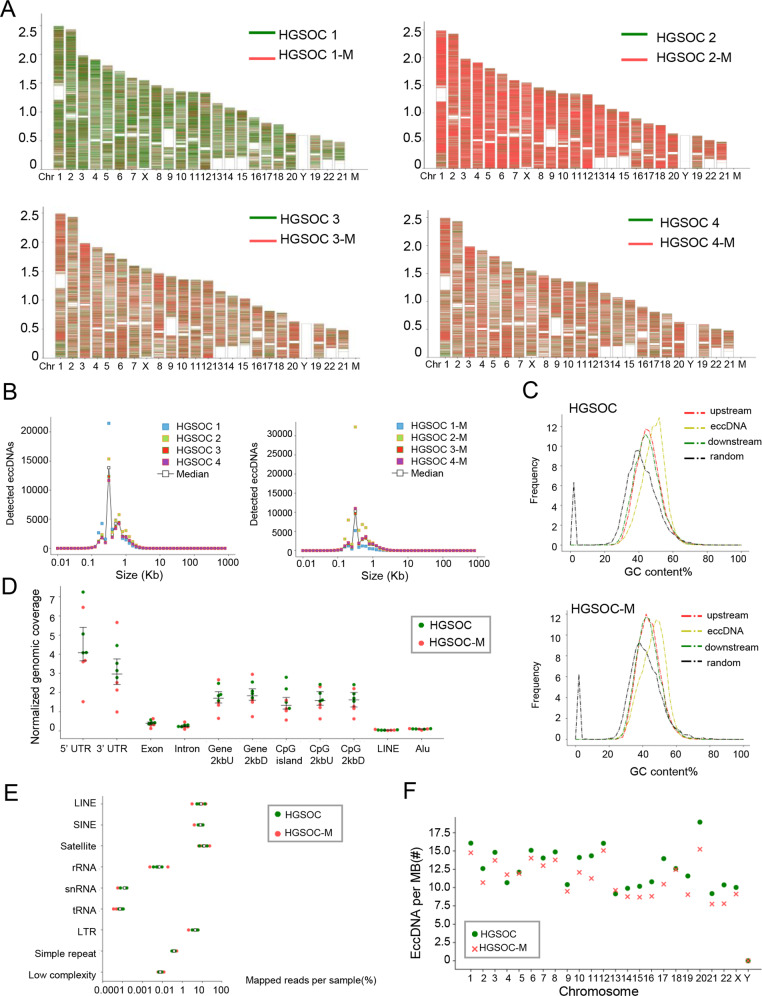
Features of eccDNAs detected in HGSOC samples. **A** The karyotype plots showing chromosomal distribution of eccDNA identified in each individual. Red, eccDNAs detected in metastatic tissues; Green, eccDNAs detected in primary tissues. **B** Size distributions of eccDNA in HGSOC (left panel) and HGSOC-M (right panel). Individuals were marked by different colors, respectively. Median was marked by white circles. **C** GC contents of eccDNA locus and regions immediately upstream and downstream from the eccDNA compared to the genomic average. HGSOC, upper panel; HGSOC-M, lower panel; Red, 1000 stretches upstream eccDNA locus (from eccDNA_start −1000 to eccDNA_start); Yellow, eccDNA (from eccDNA_start to eccDNA_end); Green, 1000 stretches downstream eccDNA locus (from eccDNA_end to eccDNA_end +1000); Black, 1000 random stretches of the genome of equivalent length as the eccDNA. **D** Genomic distributions of eccDNA in HGSOC-M (red) and HGSOC (green). Each dot represents an individual. CpG2kbD, 2 kb downstream of CpG islands; CpG2kbU, 2 kb upstream of CpG islands; Gene2kbD, 2 kb downstream of genes; Gene2kbU, 2 kb upstream of genes. **E** Repetitive regions from total mapped reads for eccDNAs derived from each sample. Red, HGSOC-M; Green, HGSOC. **F** EccDNA frequency relative to chromosome. EccDNA counts per Mb from HGSOC-M (red cross, *n* = 4) and HGSOC (green circle, *n* = 4) per chromosome.

**Fig. 3:**
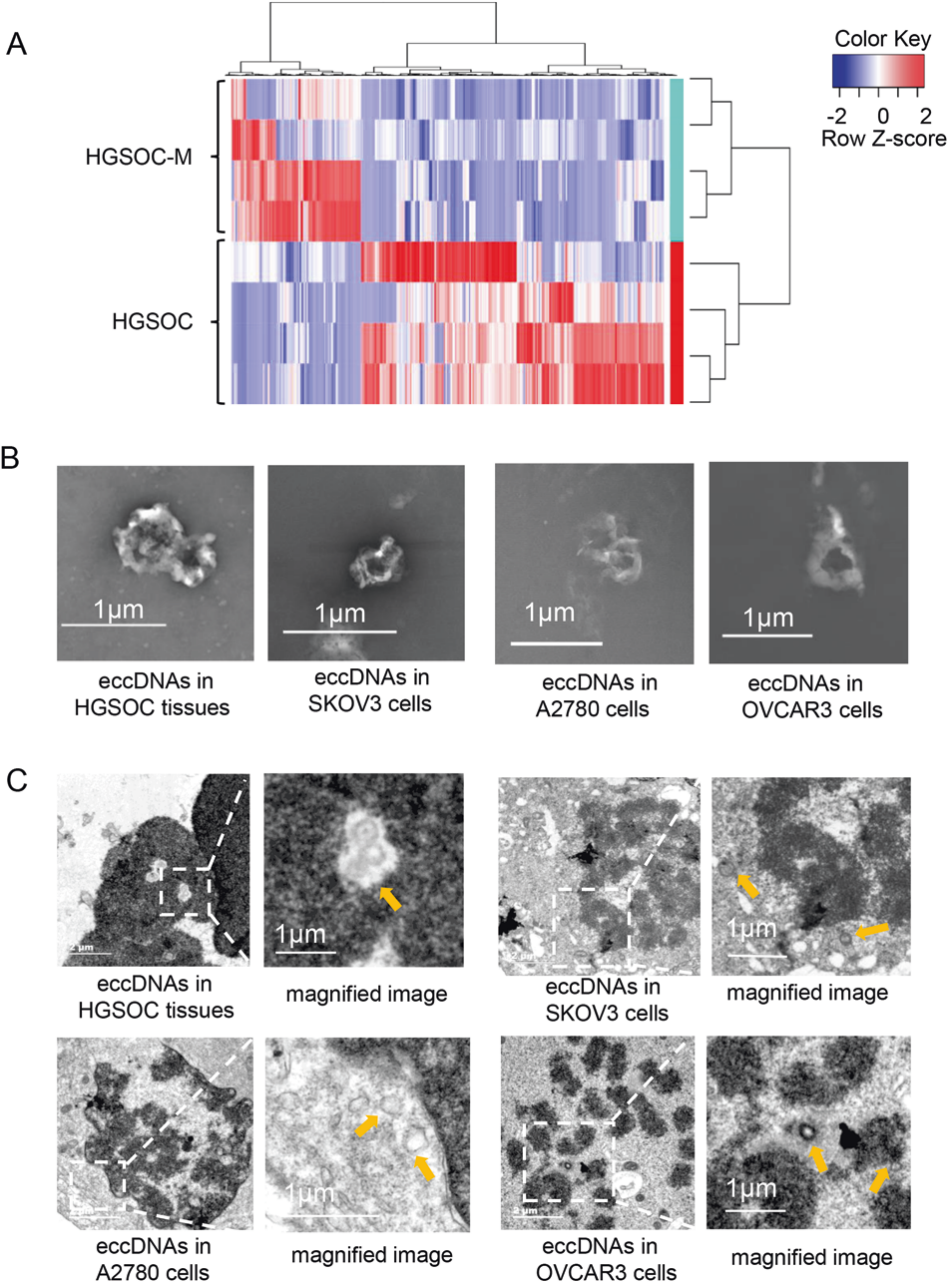
EccDNAs are differentially expressed in paired primary and metastatic tissues of HGSOC. **A** Clustered heatmap showing differentially expressed eccDNAs in paired primary tissues and metastatic tissues of four HGSOC patients. Red, up-regulation; Blue, down-regulation. **B** The SEM images of extracted eccDNAs in HGSOC tissues and ovarian cancer cell lines SKOV3, A2780 and OVCAR3. Scale bar, 1 μm. **C** The TEM images of eccDNAs in HGSOC tissues, SKOV3, A2780, and OVCAR3 cells. Scale bars, 2 μm and 1 μm. All imaging experiments were repeated at least three times, with similar results.

We then characterized eccDNA properties of the primary and metastatic tissues of HGSOC in the following aspects: expression frequency, length distribution, GC contents, and genomic distribution. Firstly, we found that these eccDNAs were derived from all the chromosomes (Fig. 2A). The expression frequencies of eccDNAs were compared in each sample, varying from 32113 to 48817 in primary tumors and 12871 to 70530 in metastatic tumors. Secondly, size distribution analysis showed that eccDNAs with the size of less than 1000 bp were the dominated subtype in HGSOC tissues (87.53% in primary tissues and 89.69% in metastatic tissues). The average size of eccDNAs of primary tissues of HGSOC was 388 bp (range from 370 to 399 bp) and the one for metastatic tissues was 379 bp (range from 371 to 419 bp), both of which peaked around 316 bp to 398 bp (Fig. 2B). Such a size distribution pattern have similarities with eccDNAs previously characterized in ovarian cancer cell line OVCAR8 [15]. Thirdly, Fig. 3C showed that there were more enrichment of GC contents in eccDNA sequences of both primary and metastatic tissues compared to other genomic regions. This indicates that rich in GC content is a common feature of eccDNAs, consistent with other reports [21]. Fourthly, we explored the possible origins of eccDNAs by mapping the eccDNAs to different genomic elements (Fig. 2D), repetitive elements (Fig. 2E) and different chromosomes (Fig. 3F). Of the both groups, we found no general correlation between gene-rich chromosomes and eccDNA formation frequency (Fig. 3F & Supplementary Fig. 1). Of note, the eccDNAs were especially enriched in both 5′ UTR region and 3′ UTR region, as well as repetitive elements such as satellites, long interspersed elements (LINEs), and short interspersed elements (SINE), suggesting that these areas rather than the gene abundance regions more preferentially generate eccDNAs in HGSOC tissues.

### EccDNAs are differentially expressed in paired primary and metastatic tissues of HGSOC

According to our sequencing results, 464 differentially expressed eccDNAs were screened out in the metastatic tissues compared to the primary tissues of HGSOC, with a cut-off standard of |FC(fold change)| ≥2 and *P* < 0.05 (Fig. 3A & Supplementary Table 2). To confirm the existence of eccDNAs, we performed scanning electron microscopy (SEM) and transmission electron microscopy (TEM) to visualize the eccDNAs in HGSOC samples as well as SKOV3, A2780 and OVCAR3 ovarian cancer cell lines. The visualization clearly showed intuitive circular structures of eccDNAs in HGSOC tissues and the three ovarian cancer cell lines (Fig. 3B, C).

### Validations of the differentially expressed eccDNAs in primary and metastatic tissues of HGSOC samples

**Fig. 4:**
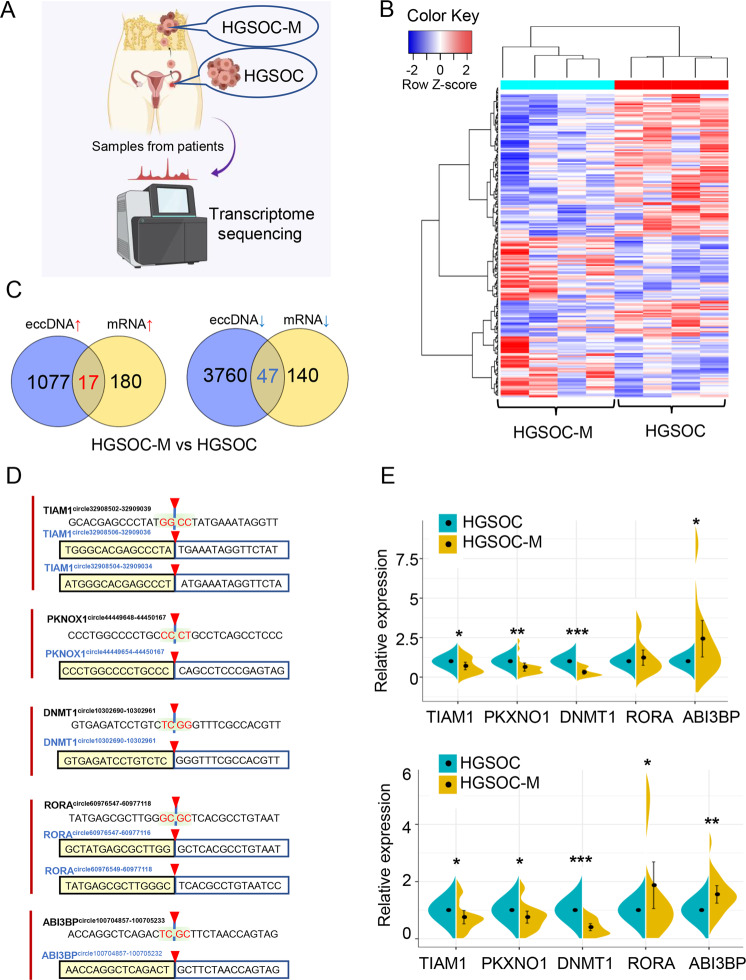
Validations of the differentially expressed eccDNAs in primary and metastatic tissues of HGSOC samples. **A** Flowchart illustrating the transcriptome analysis of paired primary and metastatic tissues of HGSOC (HGSOC 1-4 and HGSOC-M 1-4). **B** Clustered heatmap of differentially expressed mRNA in paired primary and metastatic tissues of four HGSOC patients. Red, up-regulation; Blue, down-regulation. **C** Venn diagrams showing the overlap of all differentially expressed mRNAs and eccDNAs of the same host gene. Left, up-regulated targets in both sequencing; Right, down-regulated targets in both sequencing. **D** Sanger sequencing results of PCR products of 15 bases on either side of junctions of eccDNAs derived from TIAM1, PKNOX1, DNMT1, ABI3BP and RORA,were listed in boxes, respectively. Black (upper) indicated the junction sequences of each eccDNA by Circle-Seq, with shaded (red) sequences depicting junction sites. Blue (lower) indicated the accurate circularization sites of each eccDNA based on Sanger sequencing results. **E** qPCR validations of 5 differentially expressed mRNAs (upper panel), and differentially expressed eccDNAs (lower panel) in 20 clinical HGSOC tissue samples containing 20 primary tissue samples (blue, HGSOC) and paired metastatic tissue samples (yellow, HGSOC-M). The data of the expression levels were shown as mean ± SD. *P < 0.05, **P < 0.01, ***P < 0.001.

We also determined the differentially expressed genes (DEGs) between the same primary and metastatic HGSOC tumors by RNA-sequencing analysis (Fig. 4A, B, Supplementary Table 3). We identified 219 DEGs, including 91 upregulated mRNAs and 128 downregulated mRNAs in metastatic tissues, compared to primary tissues (|FC(fold change)| ≥2 and *P* < 0.05). Besides, we analyzed the Circle-Seq data with the RNA-seq results, and found that 64 altered candidates (*P* < 0.05 in both sequencing results) were overlapped (Fig. 4C). Meanwhile, we conducted the biological process and enrichment analysis for these differentially expressed eccDNAs and mRNAs (Supplementary Fig. 2A–B). The top potential downstream functions of these candidates included metabolism regulation, angiogenesis regulation and cell death, etc. (Supplementary Fig. 3 and Supplementary Fig. 4). We selected 8 eccDNAs for further investigation according to the cancer-related functions predicted by bioinformatics analysis and the extent of eccDNA expression (Supplementary Table 4). These eccDNAs were named by their genic origin such as DNMT1^circle10302690-10302961^ [22]. To validate these predicted eccDNAs, outward PCR was performed with specific primers targeting the junction sites of each candidate. All the amplified products were separated on electrophoresis gels and appeared at the right places with the expected sizes of those candidates (Supplementary Fig. 5). In addition, we applied the Sanger sequencing to confirm the junction sites of the detected eccDNAs. The accurate genic origins of these eccDNAs are: TIAM1^circle32908504-32909034^/TIAM1^circle32908506-32909036^, PKNOX1^circle44449654-44450167^, DNMT1^circle10302690-10302961^, ABI3BP^circle100704857-100705232^ and RORA^circle60976547-60977116^/RORA^circle60976549-60977118^ (Fig. 4D). Other two candidates (VSIG10^circle118519393-118519743^, FOXO1^circle41164407-41166507^) failed to be validated due to the low expression levels. The Sanger sequencing result of PRIM2^circle57211409-57212590^ did not match the corresponding junction sites, indicating that PRIM2^circle57211409-57212590^ is not a true circular DNA. To further validate our sequencing results, qRT-PCR was performed (Fig. 4E) using another 20 pairs of primary and metastatic tissues of HGSOC (Supplementary Table 1). Among them, both DNMT1 mRNA and DNMT1^circle10302690-10302961^ are the most differentially expressed candidates at the mRNA and eccDNA levels between metastatic and primary tumors of HGSOC, thus DNMT1^circle10302690-10302961^ was chosen for further investigation.

### DNMT1^circle10302690-10302961^ is down-regulated in metastatic tumors compared to primary tumors of HGSOC

**Fig. 5:**
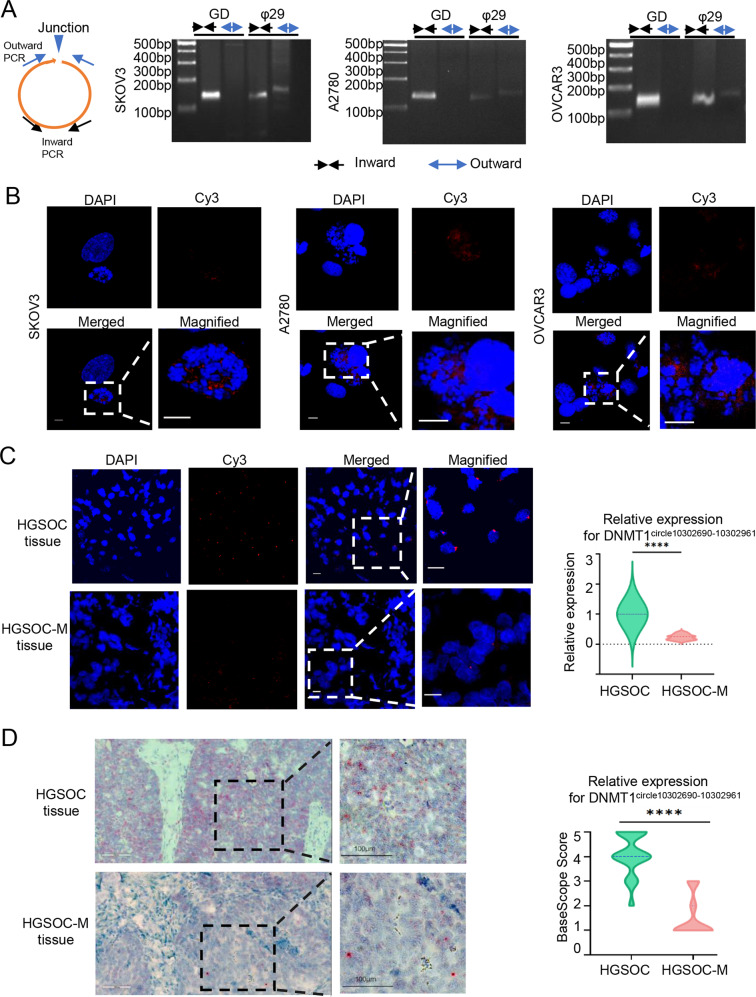
DNMT1^circle10302690-10302961^ is down-regulated in metastatic tumors compared to primary tumors of HGSOC. **A** Outward PCR (blue arrow) and inward PCR (black arrow) in genomic DNA (GD) and phi29-amplified eccDNA (φ29) samples to validate the specific junction of DNMT1^circle10302690-10302961^ in SKOV3, A2780, and OVCAR3 cells. **B** FISH assays of metaphase SKOV3, A2780, and OVCAR3 cells. The probe targeting the junction of DNMT1^circle10302690-10302961^ was labeled by Cy3. **C** The representative images of FISH assays of DNMT1^circle10302690-10302961^ in paired primary (upper) and metastatic tissues (lower) of HGSOC patients. **D** The representative images of BaseScope assays of DNMT1^circle10302690-10302961^ in paired primary (upper) and metastatic tissues (lower) of HGSOC patients. DNMT1^circle10302690-10302961^ appeared as distinct red dots, with each dot representing a single eccDNA molecule. The data were presented as mean ± SD; ****P < 0.0001.

The eccDNA DNMT1^circle10302690-10302961^ was circularized by a segment of DNMT1 gene on the reverse strand of chromosome 19 (chr19: 10302690-10302961). To verify its specific circular structure, outward PCR targeting DNMT1^circle10302690-10302961^ was performed in SKOV3, A2780 and OVCAR3 cell lines. The results showed that DNMT1^circle10302690-10302961^ was not presented in genomic DNA (Fig. 5A). The existence of DNMT1^circle10302690-10302961^ was also disclosed by Fluorescence in situ hybridization (FISH) assay using junctional specific Cy3-labeled probes in SKOV3, A2780 and OVCAR3 cells, which were arrested at metaphase spreads beforehand (Fig. 5B). The signals representing DNMT1^circle10302690-10302961^ were mainly observed off the chromosomes, ascertaining the extrachromosomal feature of this eccDNA. We then validated the DNMT1^circle10302690-10302961^ expression in formalin-fixed, paraffin-embedded (FFPE) primary and metastatic tissues of HGSOC by FISH assays and BaseScope assays (Fig. 5C, D). Both FISH assays and BaseScope assays confirmed that DNMT1^circle10302690-10302961^ was significantly down-regulated in metastatic tumors compared to its primary tumors of HSGOC.

### The decrease of DNMT1^circle10302690-10302961^ is associated with poor prognosis in HGSOC patients

**Fig. 6:**
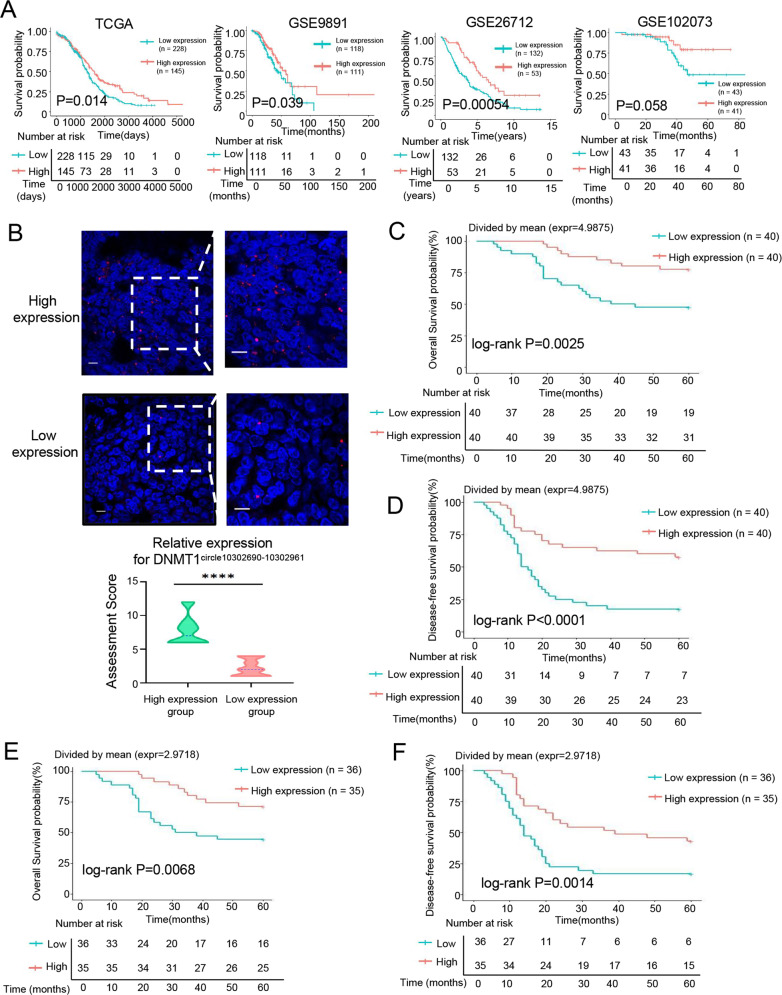
The decrease of DNMT1^circle10302690-10302961^ is associated with poor prognosis in HGSOC patients. **A** The Kaplan–Meier survival analysis of DNMT1 expression levels in ovarian cancer patients according to TCGA and GEO (GSE9891, GSE26712, GSE102073), respectively. **B** The representative images of FISH assays for DNMT1^circle10302690-10302961^ in FFPE tissues of HGSOC tumors. High expression, upper; Low expression, lower. The Kaplan–Meier survival analysis of 80 HGSOC patients stratified by DNMT1^circle10302690-10302961^ expression in FFPE tissues of primary tumors for overall survival **(C)** or disease-free survival **(D)**. The Kaplan–Meier survival analysis of 71 HGSOC patients with metastasis stratified by DNMT1^circle10302690-10302961^ expression in FFPE tissues of metastatic tumors for overall survival **(E)** or disease-free survival **(F)**. The data were presented as mean ± SD; ****P < 0.0001.

The relationship between DNMT1 expression level and the outcomes in patients with HGSOC was analyzed using four online databases (TCGA, GSE9891, GSE26712 and GSE102073). The Kaplan-Meier survival analysis showed that low expression of DNMT1 was associated with short overall survival (OS) in ovarian cancer patients (Fig. 6A). These data were consistent with our previous research that DNMT1 negatively modulates oncogenic properties of ovarian cancer [23]. As the expression of eccDNA DNMT1^circle10302690-10302961^ and DNMT1 mRNA both decreased in metastatic tumors of HGSOC, we assumed that DNMT1^circle10302690-10302961^ may also have such prognostic value. Thus, we confirmed the DNMT1^circle10302690-10302961^ expression levels in FFPE tissues of 80 HGSOC patients by FISH assays (Supplementary Table 5). All cases were divided into low or high expression group of DNMT1^circle10302690-10302961^ based on the intensity and scope of the specific probe signals (Fig. 6B). The correlation of DNMT1^circle10302690-10302961^ expression with clinicopathological factors in the 80 HGSOC patients was summarized in Table 1. DNMT1^circle10302690-10302961^ reduction was significantly associated with advanced International Federation of Gynecology and Obstetrics (FIGO) stages (*P* = 0.008), lymph node metastasis (*P* = 0.047) and postoperative visible residual disease (*P* = 0.04, in stage III-IV patients). Further Kaplan-Meier survival analysis showed that lower expression of DNMT1^circle10302690-10302961^ in primary HSGOC tumors was associated with shorter OS (Fig. 6C, *P* = 0.0025) and lower disease-free survival (DFS) rates (Fig. 6D, *P* < 0.0001). Moreover, the expression of DNMT1^circle10302690-10302961^ of the metastatic HGSOC tumors were also measured. Among the 71 samples with metastasis, patients with lower DNMT1^circle10302690-10302961^ expression also had a significantly worse OS (Fig. 6E, *P* = 0.0068) and DFS (Fig. 6F, *P* = 0.0014). In addition, we collected the primary and metastatic tissues of 25 advanced HGSOC patients (Supplementary Table 6) who received neoadjuvant chemotherapy (NACT), and measured the expression of DNMT1^circle10302690-10302961^ using FISH assays. Interestingly, the DNMT1^circle10302690-10302961^ expression in metastatic samples relative to primary tissues decreased more significantly in patients who had a partial response to NACT than those completely responded patients (Supplementary Fig. 6). Our results suggest that the decrease of DNMT1^circle10302690-10302961^ is a poor prognostic factor for HGSOC.

**Table 1:** The correlation between DNMT1^circle10302690-10302961^ expression and clinicopathological characteristics in HGSOC patients.

Characteristics	Count	DNMT1^circle10302690-10302961^ expression		*P* value
		Low	High	
Age (years)				
<50	22	12	10	0.803
≥50	58	28	30	
Differentiation				
G1 + G2	0	0	0	/
G3	80	40	40	
FIGO stage				
I + II	19	4	15	0.008
III+IV	61	36	25	
CA125 (U/mL)				
<600	40	20	20	1.000
≥600	40	20	20	
Lymph node metastasis				
negative	57	24	33	0.047
positive	23	16	7	
※Postoperative residual disease				
No visible residual disease (R0)	34	16	18	0.04
Any residual disease	27	20	7	

※The status of the residual disease of 61 patients with stages III-IV HGSOC underwent primary debulking surgery (PDS) were analyzed.

## Discussion

Advanced HGSOC are highly metastatic. This is one of the major factors leading to poor prognosis for HGSOC patients [2]. As the mechanism underlying metastasis is complicated, explorations regarding the gene expression signatures correlated to metastasis and prognosis of HGSOC is of profound significance. In this study, we expanded the current understanding of HGSOC metastasis at eccDNA levels and presented the first Circle-Seq-based eccDNA profiles of primary and metastatic tissues of HGSOC patients. Furthermore, we demonstrated that DNMT1^circle10302690-10302961^ is significantly down-regulated in metastatic tumors compared to primary tumors of HGSOC, and may potentially serve as a prognostic biomarker for HGSOC patients.

Compared with other global sequencing methods, Circle-Seq is a more sensitive method for eccDNA detection, and has been applied in yeast and healthy human somatic tissues [16, 22, 24]. Using Circle-Seq, abundant eccDNAs were detected in our HGSOC samples. The eccDNAs detected in our study were found to share some similar features (e.g., length distribution, GC contents and genomic distribution) with eccDNAs previously characterized in other reports [21, 25]. Although no significant differences were observed between the primary and metastatic tissues of HGSOC regarding the features above, the size distribution of eccDNA in all HGSOC tissues showed a distinctive peak. Compared to eccDNAs of healthy somatic tissues (peaking at 100 bp and 5 kb) and plasma of pregnant women (peaking at ~202 bp and 338 bp), eccDNA distributions in our sequencing present a peak around 316 bp to 398 bp, which might be a characteristic feature for HGSOC [22, 26, 27].

The origins of eccDNAs in HGSOC were mapped to different genomic elements. Our work has shown that eccDNAs are highly enriched in 5′ UTRs and 3′ UTRs region, where R-loop structures are formed [28, 29]. This is consistent with the previous findings that R-loop formation induces activation of the mismatch repair pathway that produce eccDNAs [25]. Besides, satellites, LINE and SINE, which arise form repetitive regions, are also the main resources of eccDNAs in HGSOC. However, the three kinds of repetitive elements produced a much smaller portion of eccDNAs in healthy somatic tissues [22]. This could be explained by the aberrant accumulation of satellites, LINE, SINE in ovarian cancer, and the active production of eccDNAs by tandem repeats [30–33].

The roles of eccDNAs have yet to be fully discovered, especially for the small size eccDNAs that are less than 1000 bp [34], of which were also the majority in our sequencing results. In recent studies, researchers found these small size eccDNAs are detectable in circulation and can serve as biomarkers due to their resistance to exonucleases digestion [21, 27, 35]. For example, the overall size of eccDNAs in plasma samples was decreased after surgery in lung cancer and ovarian cancer patients, suggesting that the size reduction of postoperative eccDNAs may act as a marker for successful tumor eradication [21]. Another study revealed that fetal-origin eccDNAs with smaller sizes could be discriminated from maternal-origin eccDNAs in serum of pregnant women, and may be a future direction for prenatal testing signature [26]. These studies have provided new insights into the clinical utilization of eccDNAs as biomarkers for disease diagnosis and prognosis.

In the present study, we identified a novel eccDNA, named DNMT1^circle10302690-10302961^, as the most significantly down-regulated eccDNA in metastatic tumors, compared to primary tumors of HGSOC. DNMT1^circle10302690-10302961^ was circularized by a segment of DNMT1 DNA. The host gene DNMT1 is a crucial regulator of genomic methylation, which mediates DNA methylation of various cancer-associated genes in regulation of metastatic propensity [36]. Loss of DNMT1 promoted metastasis in melanoma, hepatocellular carcinoma, prostate cancer and ovarian cancer [23, 37–39]. The overall survival analysis based on online database demonstrated that lower expression of DNMT1 was associated with worse OS for HGSOC patients. Interestingly, the expression of both DNMT1^circle10302690-10302961^ and DNMT1 mRNA decreased in metastatic tissues of HGSOC. Thus, we deduced that DNMT1^circle10302690-10302961^ may harbor some similar clinical value in HGSOC. Along with this idea, large amount of clinical samples were utilized to verify the clinical potential of DNMT1^circle10302690-10302961^. The decreased expression of DNMT1^circle10302690-10302961^ was associated with metastatic behaviors, as well as an adverse prognosis. It was reported that complete response to NACT was significantly associated with improved OS and DFS compared to partial or no response in HGSOC [40]. Intriguingly, we found a greater significant decrease of DNMT1^circle10302690-10302961^ expression in metastatic samples relative to primary tissues in patients who had a partial response to NACT, implying that DNMT1^circle10302690-10302961^ may also be a prospective marker to evaluate the response to chemotherapy.

In summary, we have revealed the landscape and characteristics of eccDNAs in primary and metastatic tumors of HGSOC. As the most down-regulated eccDNA in metastatic tumors of HGSOC, DNMT1^circle10302690-10302961^ is expected to be a promising biomarker to predict metastasis, prognosis, and response to NACT for patients with HGSOC. Our Circle-Sequencing results provide valuable insights into the atlas of the HGSOC eccDNA signatures. The identification of eccDNA DNMT1^circle10302690-10302961^ will contribute to advanced HGSOC treatment therapies.

## Materials and Methods

### Clinical Specimens

In this study, 4 pairs of HGSOC primary and metastatic tissues (HGSOC 1-4 and HGSOC-M 1-4) were collected in 2020 and subjected for Circle-Sequencing and transcriptome sequencing. A panel of HGSOC tissues containing 20 primary tumor samples and 20 matched metastatic tumor samples were collected in 2020 and used for validation by qRT-PCR analysis. The FFPE tissues of the same 20 HGSOC patients were also collected for validation by FISH assay and BaseScope assay. Tissue samples were immediately stored in liquid nitrogen after section and then transferred to −80 °C until use. Another panel of FFPE tissues of 80 HGSOC patients were obtained between 2013 and 2020, of whom we had complete clinical and 5-year follow-up data. The FFPE tissues of 25 advanced HGSOC patients who received NACT were obtained between 2015 and 2021.

All clinical samples above were pathologically confirmed as HGSOC, and were obtained at Women’s Hospital, School of Medicine, Zhejiang University under the approval of the Hospital Ethical Committee (IRB-20200186-R). Informed consent to use and publish clinical information for research purposes were obtained from all of the patients in accordance with the Declaration of Helsinki.

The clinical-pathological information of all patients were listed in Supplementary Table 1, Supplementary Table 5 and Supplementary Table 6.

### Circle-Sequencing analysis

The Circle-Sequencing was used to detect eccDNAs in paired primary and metastatic tissues of HGSOC patients, and the sequencing analysis was provided by CloudSeq Biotech Inc. (Shanghai, China). The procedures were referred to Circle-Seq methods previously reported and were summarized as below [22]. The samples were first incubated overnight at 50 °C supplemented with Proteinase K (Thermo Fisher, Waltham, MA, USA). The eccDNAs of samples were extracted by Plasmid Mini AX kit (A & A Biotechnology) according to the manufacturer’s instructions. Then FastDigest MssI (Thermo Scientific) and Plasmid-Safe ATP-dependent DNase (Epicenter, Madison, WI, USA) were used to remove mitochondrial circular DNA and the residual linear DNA respectively according to the manufacturer’s protocol. The purified samples were then used as templates to amplify eccDNAs by φ29 polymerase amplification (REPLI-g Midi Kit, QIAGEN, Germany). The amplification reactions were conducted at 30 °C for 46–48 h. The library preparation was conducted using NEBNext® Ultra II DNA Library Prep Kit for Illumina (New England Biolabs). The sequencing was carried out on an Illumina NovaSeq 6000 sequencer with 150 bp paired-end mode according to the manufacturer’s instructions.

Circle-map software (v1.1.4) was used to detect eccDNAs within all samples. Samtools software (v0.2) was applied to get raw soft-clipped read counts of breakpoint. The differentially expressed eccDNAs between primary and metastatic tissues of HGSOC were filtered using edgeR software (v0.6.9). EccDNAs were annotated by Bedtools software (v2.27.1). The GO and Pathway enrichment analysis were performed based on the differentially expressed eccDNA-associated genes.

### RNA-Seq analysis

Total RNA was extracted from paired primary and metastatic tissues of the same four HGSOC patients in Circle-Seq analysis. The library preparation and RNA-sequencing on an Illumina HiSeq 4000 sequencer were carried out at CloudSeq Biotech Inc. (Shanghai, China). High quality clean reads were aligned to the reference genome (UCSC hg19) with hisat2 software (v2.0.4). Based on gene level FPKM acquired by cuffdiff software, fold change and *P* value were calculated to identify the differentially expressed mRNA. The GO and Pathway enrichment analysis were further performed according to the filtered differentially expressed mRNA.

### Cell culture

Human ovarian cancer cell lines SKOV3, A2780, and OVCAR3 were purchased from the American Type Culture Collection (Manassas, MA, USA) and cultured as described [41, 42].

### Scanning electron microscopy (SEM)

EccDNA samples of HGSOC tissues, SKOV3, A2780 and OVCAR3 cells were prepared using the Plasmid Mini AX kit (A & A Biotechnology). The SEM analysis was conducted at Center of Cryo-Electron Microscopy (CCEM), Zhejiang University using a field-emission scanning electron microscopy (Nova Nano 450).

### Transmission electron microscopy (TEM)

HGSOC tissues, SKOV3, A2780 and OVCAR3 cells were prepared for TEM analysis in the routine manner. The TEM analysis was conducted at CCEM, Zhejiang University using a cryo-transmission electron microscopy (Tecnai G2 Spirit) at 120KV.

### Outward PCR and inward PCR

The primers for outward PCR were designed across the specific junction sites of each eccDNA candidate. The φ29 amplified samples or genomic DNA were used as templates and the PCR reactions were carried out under standard PCR conditions. Inward PCR was used as a positive control in both linear and circular DNA templates. Primers for outward PCR were designed by CloudSeq Biotech Inc. (Shanghai, China). The primer sequences were listed in Supplementary Table 7.

### RNA extraction and qRT-PCR analysis

Total RNA was extracted from tissues using Trizol reagent (Invitrogen, New York, USA), and RNA was then reverse transcribed into cDNA using the PrimeScript RT reagent kit (TaKaRa, Japan). QRT-PCR was carried out using TB Green Premix Ex Taq kit (TaKaRa, Japan). For mRNA validation, GAPDH was used as the internal control. For eccDNA validation, plasmid pGEX-5X-2 was added to the samples prior to eccDNA purification and used as the internal control [22]. The relative expression levels were analyzed using the 2^−ΔΔCt^ method. The primers for qRT-PCR analysis were provided in Supplementary Table 7.

### Fluorescence in situ hybridization (FISH) assay

FISH assays were performed in SKOV3, A2780 and OVCAR3 cells, as well as FFPE tissues of HGSOC patients. Cy3-labeled probe specific to the junction sites of DNMT1^circle10302690-10302961^ was designed at RiboBio (Guangzhou, China). The probe sequences were available upon request. The cells were first treated with 0.2 μg/mL colcemid for 4 hours to arrest the cells at metaphase stage. The FFPE tissues of patients diagnosed as HGSOC were deparaffinized and rehydrated beforehand. The signals of DNMT1^circle10302690-10302961^ in prepared cells or tissues were detected using Fluorescent In Situ Hybridization Kit (RiboBio, Guangzhou, China) following the manufacturer’s instructions. The representative images were captured by a laser confocal microscope (TCS SP2 AOBS) at 60× magnification. The relative expression levels of DNMT1^circle10302690-10302961^ were evaluated and scored based on the intensity and scope of specific signals under blinded circumstance.

### BaseScope assay

The expression of DNMT1^circle10302690-10302961^ in FFPE tissue of HGSOC was evaluated by BaseScope Assay (Advanced Cell Diagnostics, Newark, CA, USA). A BaseScope probe specifically targeting the junction sites of DNMT1^circle10302690-10302961^ was designed by Advanced Cell Diagnostics. FFPE tissues of HGSOC patients were prepared following the manufacturer’s instruction. BaseScope assays were conducted using a BaseScope Detection Reagent Kit-RED (Advanced Cell Diagnostics, Newark, CA, USA) according to the manufacturer’s instructions. The BaseScope Fast RED reagent (Advanced Cell Diagnostics, Newark, CA, USA) was used to detect the signals. At 20× magnification, the relative expression levels of DNMT1^circle10302690-10302961^ were evaluated and scored according to the manufacturer’s instructions.

### Statistical analysis

All statistical plots and analyses were executed on GraphPad Prism 8.0 and SPSS 22.0 software in this study. All the data were normally distributed. Variance was similar between the groups that were being statistically compared. Significance was determined using paired Student’s *t* test, Log-Rank test (for Kaplan-Meier curves), and χ^2^ test (for clinicopathological analysis) where appropriate. Differences were statistically significant at *P < 0.05, **P < 0.01, ***P < 0.001 and ****P < 0.0001.

## Supplementary information

reproducibility checklist

Supplementary figures 1-6

Supplementary Table 1

Supplementary Table 2

Supplementary Table 3

Supplementary Table 4

Supplementary Table 5

Supplementary Table 6

Supplementary Table 7

## Data Availability

All data needed to evaluate the conclusions in the paper are present in the paper and/or the Supplementary Materials. Additional data related to this paper can be requested from the corresponding authors.
