# Supplementary material for: Global characterization of extrachromosomal circular DNAs in advanced high grade serous ovarian cancer
Source: Cell Death Dis. 2022 Apr 13;13(4):342. doi: 10.1038/s41419-022-04807-8 (PMC9007969; doi:10.1038/s41419-022-04807-8)
Supplement: Supplementary file 2 — Supplementary figures 1-6 [file 41419_2022_4807_MOESM2_ESM.pdf]

Supplementary Fig. 1-6

for

Global characterization of extrachromosomal circular DNAs in advanced high grade serous ovarian cancer

Yixuan Cen, Yifeng Fang, Yan Ren, Shiyuan Hong *, Weiguo Lu * and Junfen Xu *

*Corresponding authors: Dr. Junfen Xu, email: xjfzu@zju.edu.cn

Prof. Weiguo Lu, e-mail: lbwg@zju.edu.cn

Prof. Shiyuan Hong, email: hongshiyuan@cqmu.edu.cn

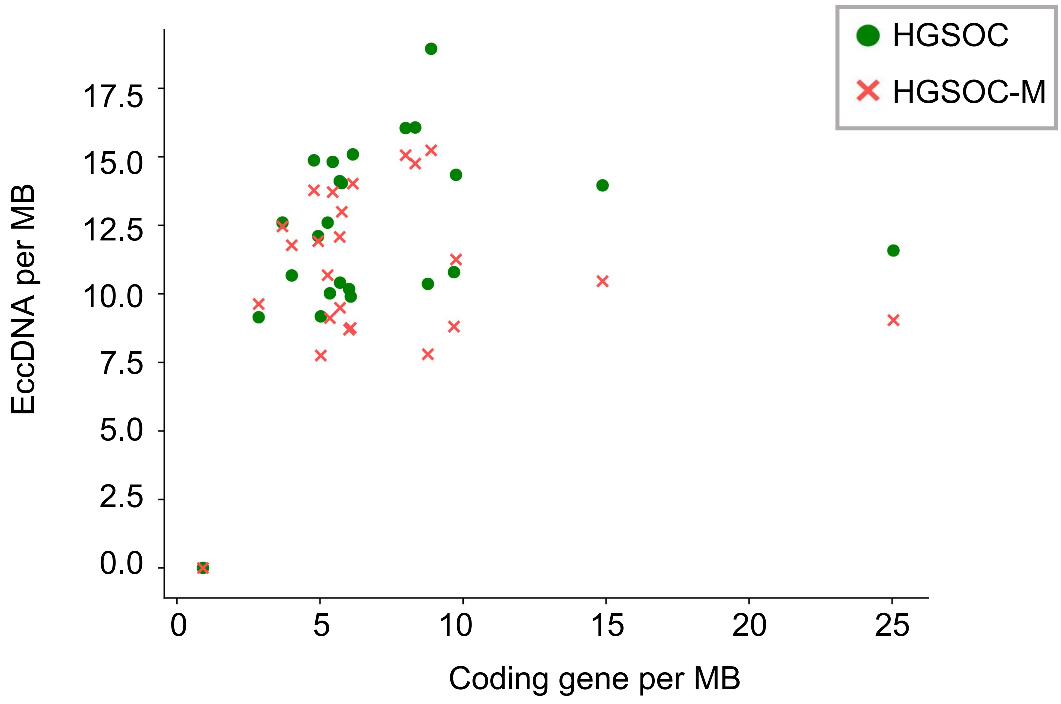

Supplementary Fig. 1 EccDNA counts per MB from HGSOC-M tissues (red cross, n=4) and HGSOC tissues (green dot, n=4) per coding genes per MB.

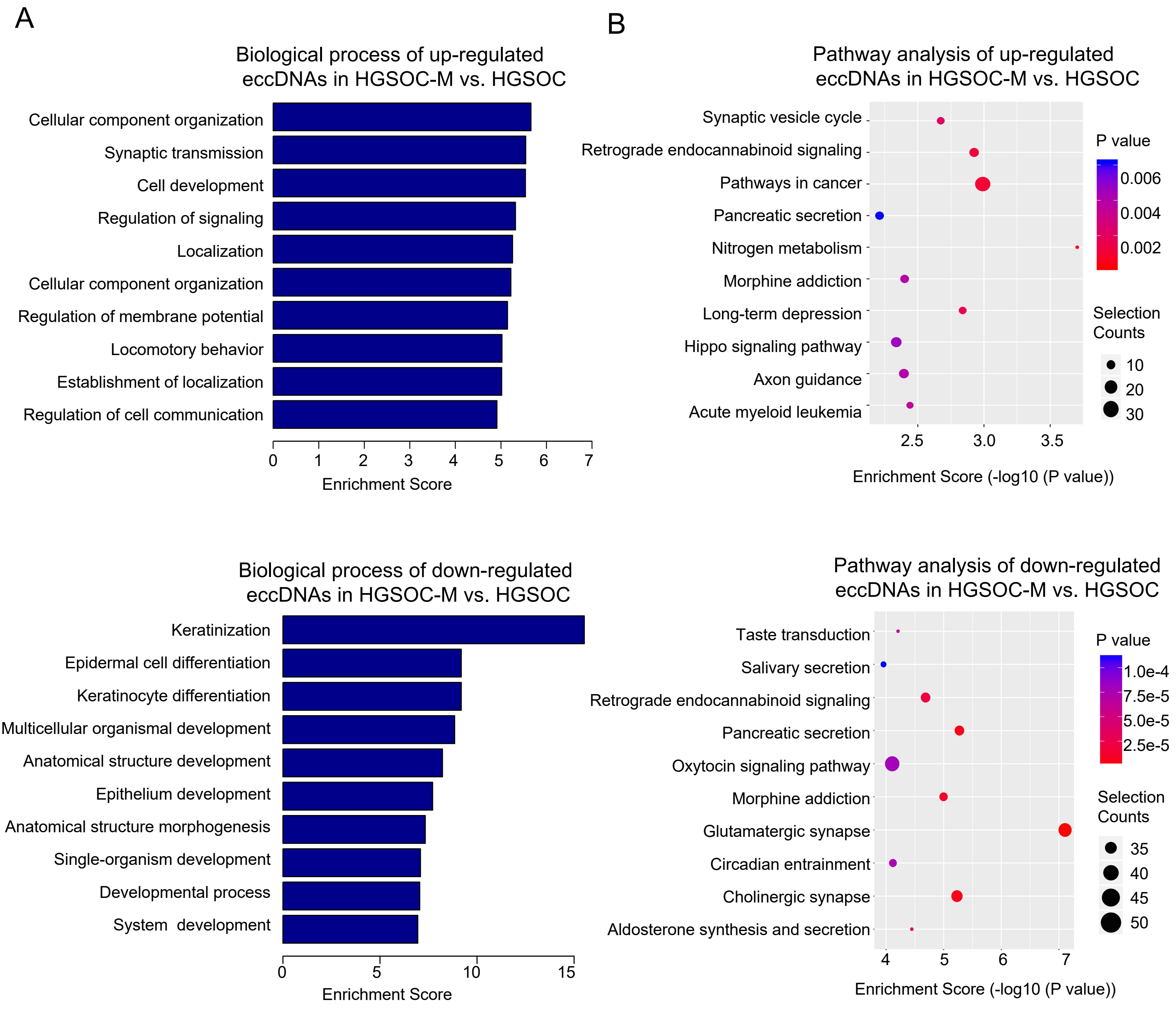

Supplementary Fig. 2 **A** The biological process analysis of differentially expressed eccDNAs between HGSOC-M and HGSOC group. Upper, differentially up-regulated in HGSOC-M; Lower, differentially down-regulated in HGSOC-M; **B** The enrichment pathway analysis of differentially expressed eccDNAs between HGSOC-M and HGSOC group. Upper, differentially up-regulated in HGSOC-M; Lower, differentially down-regulated in HGSOC-M;

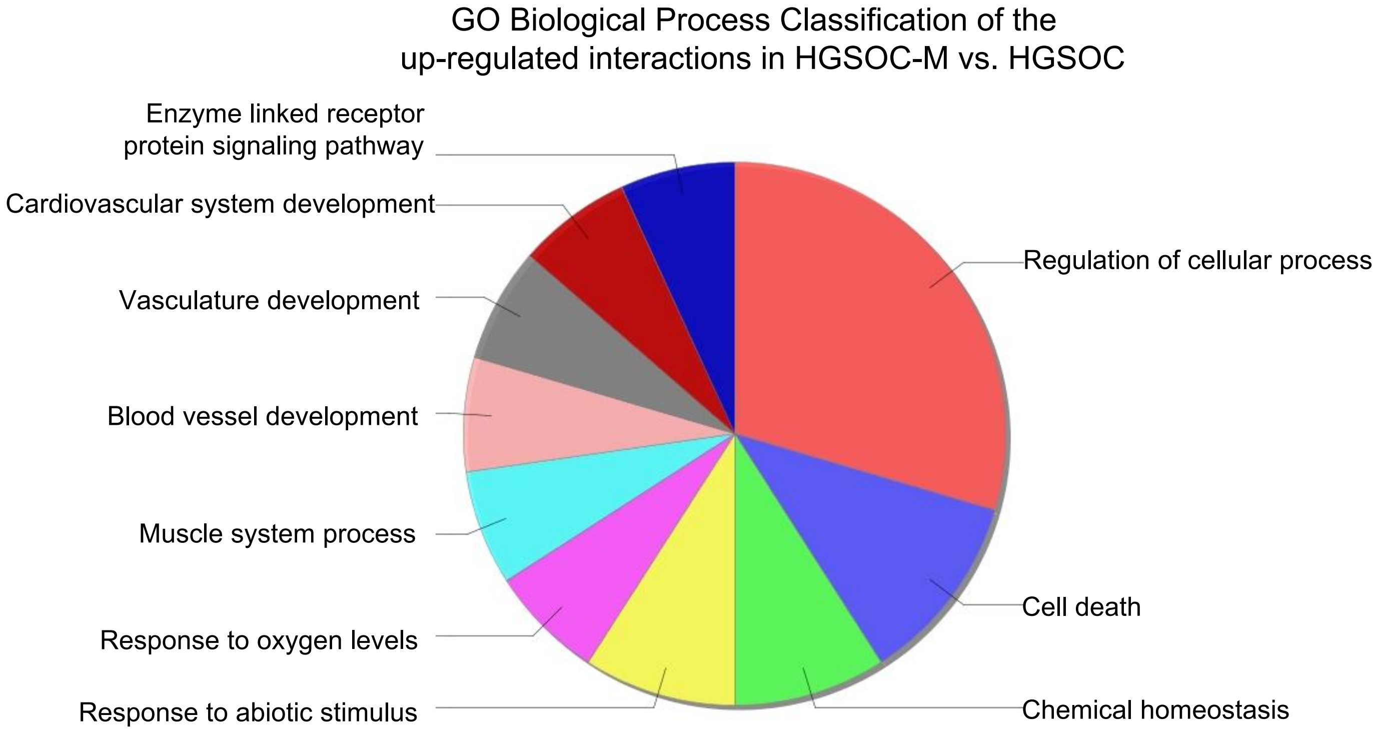

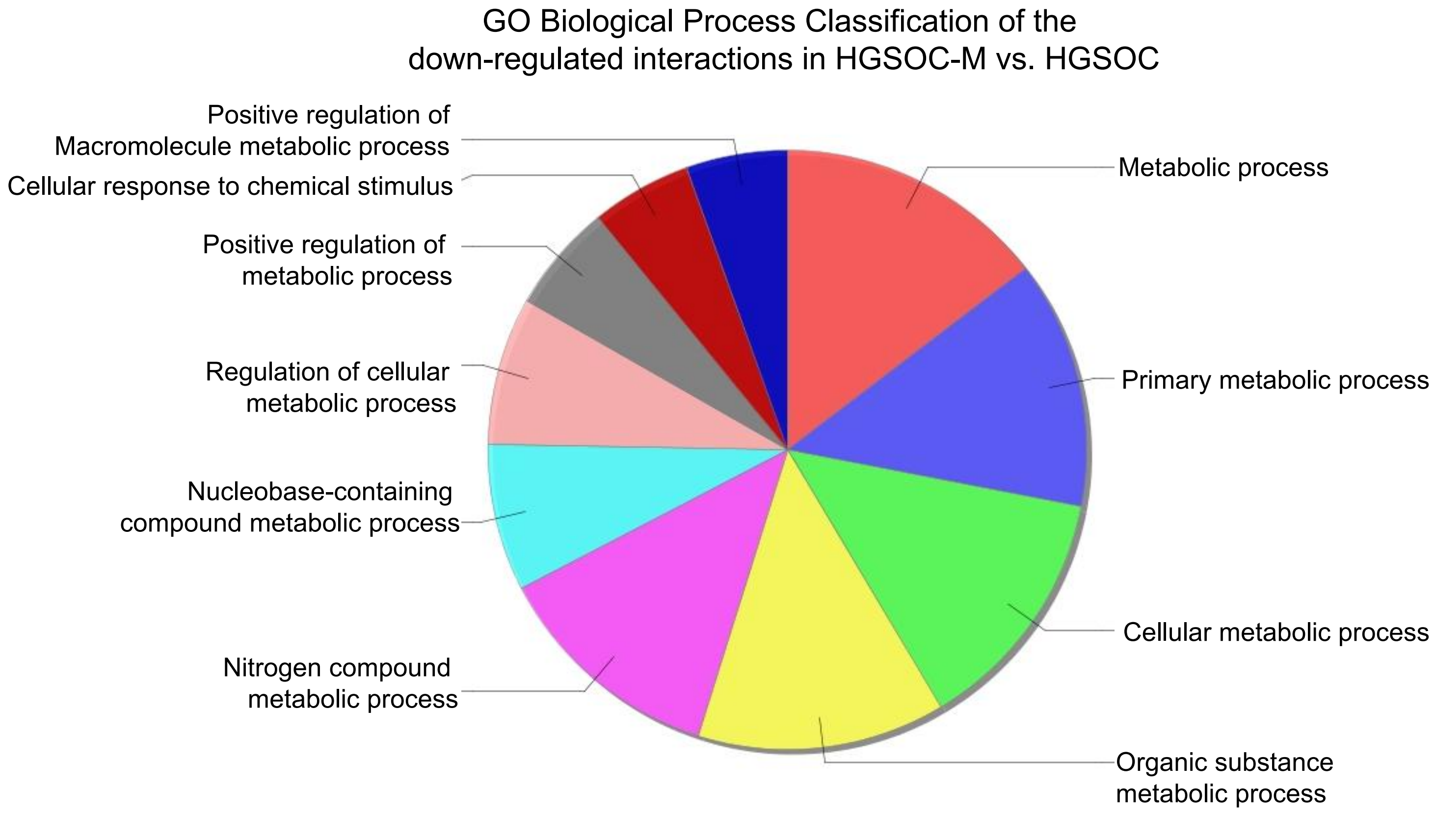

Supplementary Fig. 3 The classification of GO biological process of the overlap targets of Circle-Seq and RNA-Seq. Upper, differentially up-regulated in HGSOC-M; Lower, differentially down-regulated in HGSOC-M.

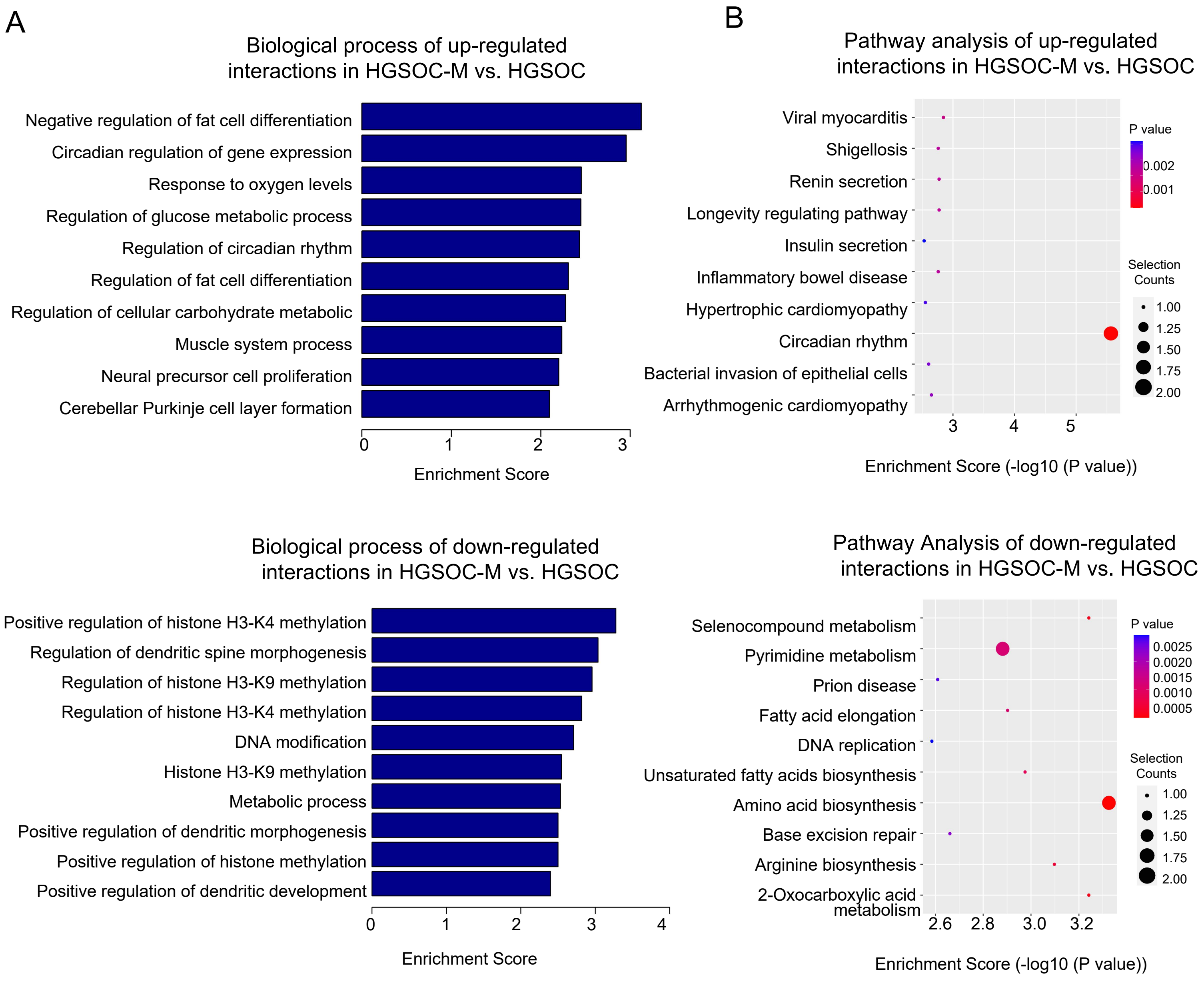

Supplementary Fig. 4 (A) The biological process analysis of overlap targets of Circle-Seq and RNA-Seq. Upper, differentially up-regulated in HGSOC-M; Lower, differentially down-regulated in HGSOC-M; **(B)** The enrichment pathway analysis of the overlap targets of Circle-Seq and RNA-Seq. Upper, differentially up-regulated in HGSOC-M; Lower, differentially down-regulated in HGSOC-M;

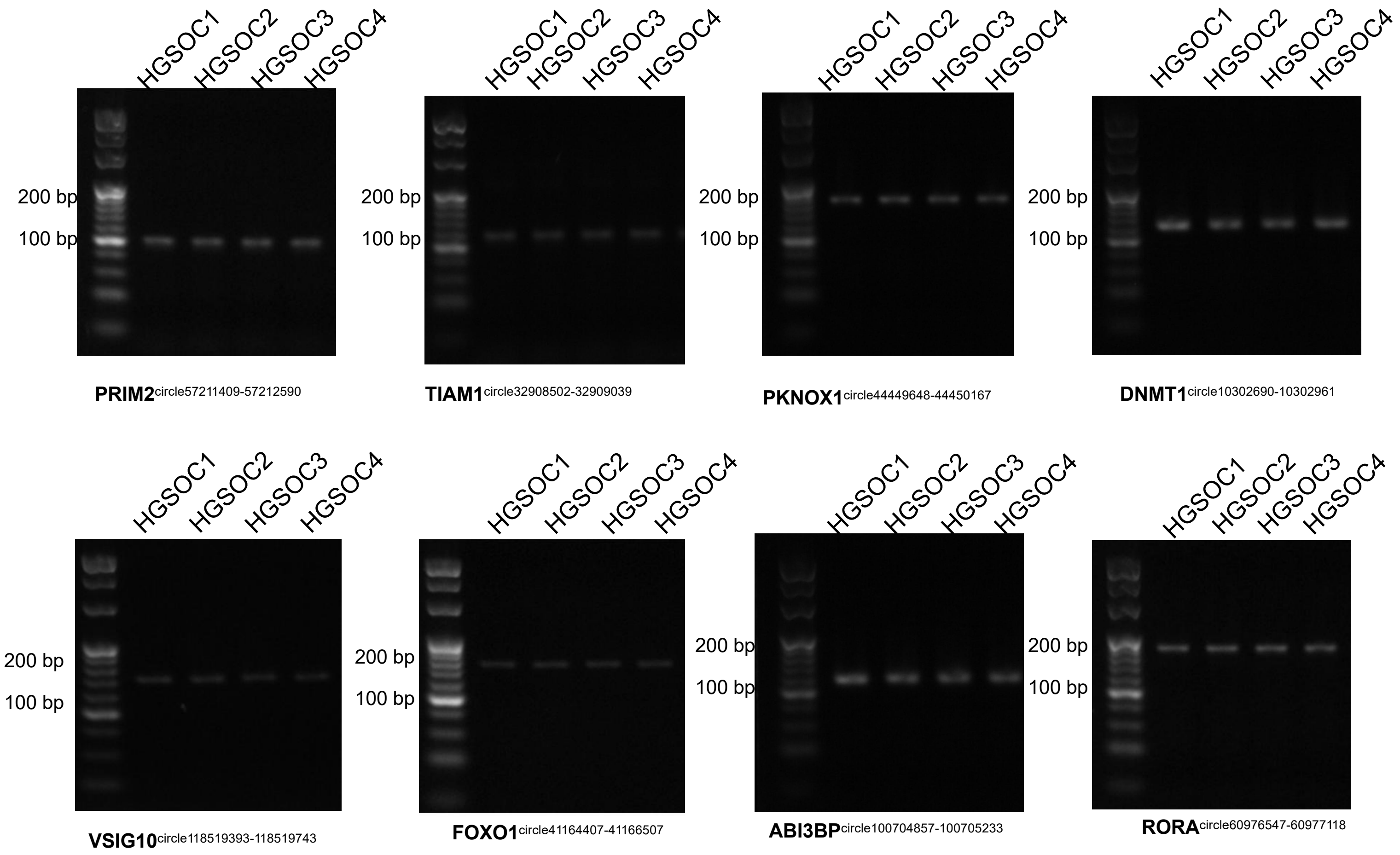

Supplementary Fig. 5 Gel electrophoresis analysis for a validated subset (n = 8) of eccDNAs by outward PCR using HGSOC1-4 tissues. EccDNAs were named according to gene content.

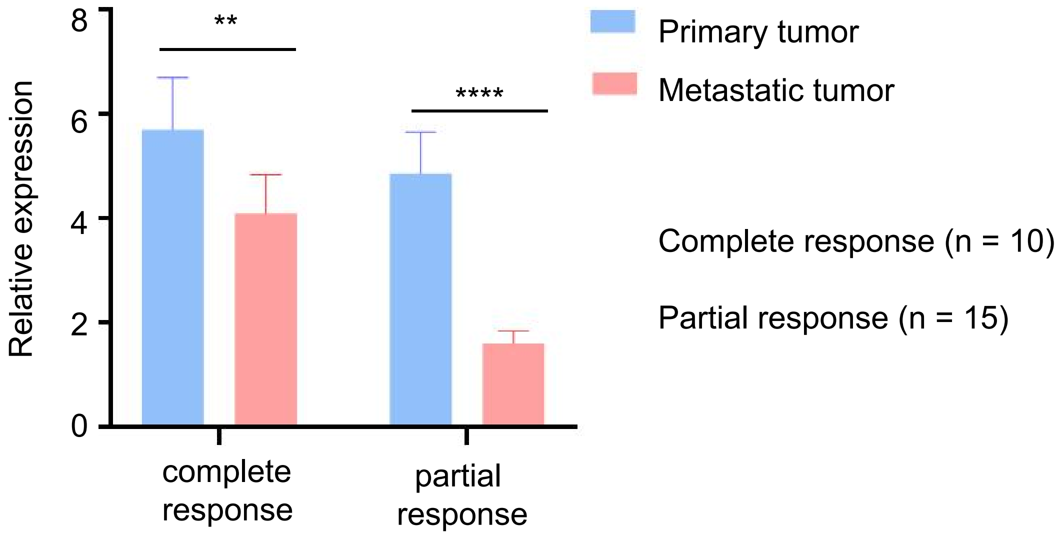

Supplementary Fig. 6 The DNMT1$^{circle10302690-10302961}$ expression of metastatic and primary tumors in 25 HGSOC patients who received NACT. The data were shown as mean ± SD. ** P < 0.01, **** P < 0.0001.
