# Supplementary material for: Global characterization of extrachromosomal circular DNAs in advanced high grade serous ovarian cancer
Source: Cell Death Dis. 2022 Apr 13;13(4):342. doi: 10.1038/s41419-022-04807-8 (PMC9007969; doi:10.1038/s41419-022-04807-8)
Supplement: Supplementary file 3 — Supplementary Table 1 [file 41419_2022_4807_MOESM3_ESM.pdf]

Supplementary Table 1.

Information for specimens

The paired primary and metastatic tissues of HGSOC were collected from patients who underwent surgical cytoreduction. All patients did not receive any radiotherapy or chemotherapy before operation.

For eccDNA-seq/mRNA-seq

Pathological diagnosis	Sample No.	age	stage	Primary site	Metastatic site
HGSOC	1	48	IIIC	Left ovary	Metastatic tumor at Omentum
HGSOC	2	51	IIIC	Right ovary	Metastatic tumor at Omentum
HGSOC	3	50	IVB	Left ovary	Metastatic tumor at spleen
HGSOC	4	82	IIIA1i	Left ovary	Metastatic tumor at Rectum

For validation

Pathological diagnosis	Sample No.	age	stage	Primary site	Metastatic site
HGSOC	1	50	IIIC	Right ovary	Metastatic tumor at Omentum
HGSOC	2	55	IIIA	Left ovary	Lymph nodes of left pelvic
HGSOC	3	50	IIIB	Right ovary	Metastatic tumor at Peritoneum
HGSOC	4	71	IIIC	Left ovary	Metastatic tumor at omentum
HGSOC	5	68	IIIC	Right ovary	Metastatic tumor at omentum
HGSOC	6	59	IVB	Left ovary	Metastatic tumor at left paracolic sulcus

HGSOC	7	57	IIIC	Left ovary	Metastatic tumor at rectum surface
HGSOC	8	49	IIB	Left ovary	Metastatic tumor at sigmoid surface
HGSOC	9	71	IIIA1（ii）	Left ovary	Metastatic tumor at sigmoid surface
HGSOC	10	51	IIIB	Right ovary	Metastatic tumor at rectum surface
HGSOC	11	52	IIIC	Right ovary	Metastatic tumor at mesocolon transversum
HGSOC	12	42	IVB	Left ovary	Metastatic tumor at Omentum
HGSOC	13	51	IIIC	Left ovary	Metastatic tumor at Omentum
HGSOC	14	50	IIIC	Left ovary	Metastatic tumor at mesenteriolum
HGSOC	15	58	IIIC	Left ovary	Metastatic tumor at paracolonic sulcus peritoneum
HGSOC	16	50	IIIB	Right ovary	Metastatic tumor at omentum
HGSOC	17	58	IIIC	Left ovary	Metastatic tumor at colon ascendens
HGSOC	18	62	IIIC	Right ovary	Metastatic tumor at colon descendens
HGSOC	19	48	IIIC	Left ovary	Metastatic tumor at omentum
HGSOC	20	50	IIIC	Right ovary	Metastatic tumor at ileocecal junction
