# Supplementary material for: Global characterization of extrachromosomal circular DNAs in advanced high grade serous ovarian cancer
Source: Cell Death Dis. 2022 Apr 13;13(4):342. doi: 10.1038/s41419-022-04807-8 (PMC9007969; doi:10.1038/s41419-022-04807-8)
Supplement: Supplementary file 6 — Supplementary Table 4 [file 41419_2022_4807_MOESM6_ESM.pdf]

Supplementary Table 4.

The eight eccDNA candidates selected from the overlap of Circle-Seq and transcriptome sequencing.

Chr	Start	End	Size (bp)	Gene present	mRNA HGSOC-M vs HGSOC	eccDNA HGSOC-M vs HGSOC
Chr21	32908502	32909039	538	TIAM1	↓	↓
Chr6	57211409	57212590	1182	PRIM2	↓	↓
Chr21	44449648	44450167	520	PKNOX1	↓	↓
Chr19	10302690	10302961	271	DNMT1	↓	↓
Chr12	118519393	118519743	351	VSIG10	↓	↓
Chr3	100704857	100705233	377	ABI3BP	↑	↑
Chr15	60976547	60977118	572	RORA	↑	↑
Chr13	41164407	41166507	2101	FOXO1	↑	↑
