# Supplementary material for: Global characterization of extrachromosomal circular DNAs in advanced high grade serous ovarian cancer
Source: Cell Death Dis. 2022 Apr 13;13(4):342. doi: 10.1038/s41419-022-04807-8 (PMC9007969; doi:10.1038/s41419-022-04807-8)
Supplement: Supplementary file 7 — Supplementary Table 5 [file 41419_2022_4807_MOESM7_ESM.pdf]

Supplementary Table 5

Information of 80 HGSOC patients with complete 5-year follow up used for OS and DFS analysis.

Case No.	age (years)	FIGO stage	Grade	CA125 (U/mL)	No visible residual disease (R0) (for FIGO Ⅲ-Ⅳ）	lymph node metastasis	DFS time (months)	OS time (months)
1	58	IIB	G3	663.8	/	(-)	60	60
2	53	IIB	G3	35.3	/	(-)	60	60
3	52	IIIC	G3	725.8	no	(-)	20	26
4	61	IIIC	G3	1097	no	(-)	14	19
5	49	IIIC	G3	956.6	yes	(-)	21	45
6	69	IV	G3	659	yes	(-)	5	7
7	58	IIIC	G3	2006	yes	(+)	11	52
8	71	IIIC	G3	108.5	yes	(-)	18	60
9	57	IIB	G3	702.9	/	(-)	60	60
10	54	IIIC	G3	1872	no	(-)	17	60
11	62	IIB	G3	318	/	(-)	14	60
12	61	IIIC	G3	4374	yes	(+)	20	60
13	34	IC1	G3	28.1	/	(-)	60	60
14	55	IIIC	G3	364.8	no	(-)	33	60
15	66	IIIC	G3	363.6	no	(+)	14	29
16	50	IIIC	G3	466.8	no	(+)	20	38
17	57	IIA	G3	563.3	/	(-)	60	60
18	62	IIC	G3	363.1	/	(-)	14	38
19	62	IIC	G3	44.4	/	(-)	60	60
20	63	IIIC	G3	209.4	no	(+)	60	60
21	45	IIIC	G3	3666.7	yes	(+)	60	60
22	65	IIIC	G3	409.4	yes	(-)	60	60
23	77	IIIC	G3	103.9	yes	(-)	12	60
24	43	IIIC	G3	3940	no	(+)	24	60
25	49	IIIC	G3	2456	yes	(+)	13	60
26	49	IIIA2	G3	74.9	yes	(-)	60	60
27	50	IIIC	G3	770.3	no	(-)	39	60
28	60	IIA	G3	13	/	(-)	60	60
29	49	IIA	G3	41.63	/	(-)	60	60
30	51	IIIC	G3	1213	yes	(+)	59	60
31	39	IV	G3	884.9	yes	(-)	60	60
32	55	IIIC	G3	717.4	no	(-)	60	60
33	55	IIIC	G3	1139	yes	(-)	19	60
34	50	IIIC	G3	196.9	yes	(+)	60	60
35	39	IIIC	G3	3159	no	(+)	29	60
36	56	IIIc	G3	9868	yes	(-)	12	60

37	62	IA	G3	67.5	/	(-)	60	60
38	64	IIIC	G3	148.2	yes	(-)	60	60
39	54	IC	G3	18.9	/	(-)	60	60
40	59	IIIB	G3	485.1	yes	(-)	60	60
41	57	IIIC	G3	1374	no	(-)	12	60
42	47	IIIC	G3	202.6	yes	(-)	60	60
43	57	IIIA	G3	369.1	yes	(-)	60	60
44	49	IIIC	G3	258.1	no	(+)	17	60
45	47	IIIC	G3	9744	yes	(-)	60	60
46	56	IIIC	G3	264.2	yes	(-)	60	60
47	55	IIC	G3	12.5	/	(-)	60	60
48	46	IIA	G3	182.4	/	(-)	60	60
49	59	IIIC	G3	2215	no	(-)	60	60
50	68	IIA	G3	36.9	/	(-)	60	60
51	58	IC	G3	228.3	/	(-)	60	60
52	54	IIB	G3	1054	/	(-)	22	60
53	41	IIIC	G3	600	yes	(-)	60	60
54	41	IIC	G3	805.8	/	(-)	26	60
55	52	lllC	G3	1000	no	(-)	13	24
56	45	IIIB	G3	335.8	no	(+)	13	31
57	62	IIIC	G3	342.6	yes	(-)	19	30
58	71	IIB	G3	20.7	/	(-)	11	24
59	52	IIIC	G3	1996	no	(+)	10	23
60	78	IIIC	G3	1665	no	(-)	14	20
61	49	IIIA1	G3	211.5	yes	(+)	36	41
62	51	IIIC	G3	1330	no	(+)	4	6
63	69	IIIC	G3	153.3	yes	(-)	7	17
64	43	IIIC	G3	217.2	no	(-)	8	16
65	62	IIIC	G3	216.8	yes	(+)	8	18
66	63	IIIC	G3	1745	yes	(+)	16	19
67	49	IIIC	G3	140.5	no	(+)	6	10
68	48	IIIC	G3	1822	no	(+)	10	19
69	53	IVB	G3	1245	no	(+)	12	35
70	51	IIIC	G3	204.3	yes	(-)	22	32
71	65	IIIC	G3	622.3	no	(-)	9	19
72	63	IIIC	G3	727.6	no	(-)	12	19
73	45	IIIB	G3	89.5	yes	(-)	3	5
74	56	IIIC	G3	1894	no	(+)	11	23
75	64	IIIC	G3	546.6	no	(-)	9	17
76	67	IIIC	G3	4643	yes	(-)	12	34
77	63	IIIC	G3	2830	yes	(-)	18	60
78	61	IIIC	G3	62.2	yes	(-)	60	60
79	43	IIIC	G3	832.6	yes	(-)	8	60
80	50	IIIC	G3	812.3	yes	(+)	48	60

(-) represents negative lymph node metastasis;
(+) represents positive lymph node metastasis.
