# Supplementary material for: Global characterization of extrachromosomal circular DNAs in advanced high grade serous ovarian cancer
Source: Cell Death Dis. 2022 Apr 13;13(4):342. doi: 10.1038/s41419-022-04807-8 (PMC9007969; doi:10.1038/s41419-022-04807-8)
Supplement: Supplementary file 8 — Supplementary Table 6 [file 41419_2022_4807_MOESM8_ESM.pdf]

Supplementary Table 6

Information of 25 advanced HGSOC patients received neoadjuvant chemotherapy (NACT).

Case No.	age (years)	Pathological diagnosis	FIGO stage	NACT
1	64	HGSOC	IIIC	1
2	58	HGSOC	IIIC	1
3	51	HGSOC	IV	1
4	63	HGSOC	IIIC	1
5	49	HGSOC	IV	1
6	66	HGSOC	IIIB	1
7	48	HGSOC	IV	1
8	41	HGSOC	IVA	1
9	72	HGSOC	IV	1
10	30	HGSOC	IV	1
11	61	HGSOC	IIIC	1
12	61	HGSOC	IVB	1
13	59	HGSOC	IIIC	1
14	71	HGSOC	IIIC	1
15	68	HGSOC	IV	1
16	71	HGSOC	IIIC	1
17	76	HGSOC	IVB	1
18	31	HGSOC	IVA	1
19	55	HGSOC	IVB	1
20	72	HGSOC	IVB	1
21	74	HGSOC	IV	1
22	47	HGSOC	IV	1
23	63	HGSOC	IVB	1
24	73	HGSOC	IV	1
25	63	HGSOC	IV	1

1 represents that the patient has received NACT treatment.
