# Supplementary material for: Global characterization of extrachromosomal circular DNAs in advanced high grade serous ovarian cancer
Source: Cell Death Dis. 2022 Apr 13;13(4):342. doi: 10.1038/s41419-022-04807-8 (PMC9007969; doi:10.1038/s41419-022-04807-8)
Supplement: Supplementary file 9 — Supplementary Table 7 [file 41419_2022_4807_MOESM9_ESM.pdf]

Supplementary Table 7

Primers in this study.

primers		
Gene	Primer	Sequence(5' to 3')
pGEX-5X-2	Forward	GGGCTGGCAAGCCACGTTTGGTG
	Reverse	CCGGGAGCTGCATGTGTCAGAGG
GAPDH	Forward	TCACCACCATGGAGAAGGC
	Reverse	GCTAAGCAGTTGGTGGTGCA
TIAM1 (outward PCR)	Forward	GGTGAGCAAGGAGTGAGGAG
	Reverse	TGCCTTATGCCTCAGTTTCC
PRIM2 (outward PCR)	Forward	TCCTCACAACAGCTCCATGA
	Reverse	GGGAACTTGGAGGAAAGAGG
PKNOX1 (outward PCR)	Forward	GTCTGTGGACAGCACAGAGG
	Reverse	CGGATAACGAGGTCAGGAGA
DNMT1 (outward PCR)	Forward	GGTTCAAGGCTGCAGTAAGC
	Reverse	AGATGGGCAGATCACCTGAG
VSIG10 (outward PCR)	Forward	TGCCTGTAATCCCAGCTACC
	Reverse	TGTGGCTCCCTCAATACACA
FOXO1 (outward PCR)	Forward	AATCTCCAGCACTCCGCTTA
	Reverse	TGCATCATTGGGCTTAGATG
ABI3BP (outward PCR)	Forward	AATCTCCAGCACTCCGCTTA
	Reverse	TGCATCATTGGGCTTAGATG
RORA (outward PCR)	Forward	AGGCTTGCACATTGACTCCT
	Reverse	ACGTCATGATCCACCCATCT
TIAM1	Forward	GATCCACAGGAACTCCGAAGT
	Reverse	GCTCCCGAAGTCTTCTAGGGT
PKNOX1	Forward	ATAGACAGCTATCAAGATGGGCA
	Reverse	GCATCGGGTTCAGAGCAGTTT
DNMT1	Forward	AGGCGGCTCAAAGATTTGGAA
	Reverse	GCAGAAATTCGTGCAAGAGATTC
ABI3BP	Forward	CAAATGCAACATGCTCTCCAGT
	Reverse	TTGGCCTTTTACCTTTTGGCA
RORA	Forward	ACTCCTGTCCTCGTCAGAAGA
	Reverse	CATCCCTACGGCAAGGCATTT
